# Development and validation of an instrument to verify vaccination hesitancy against COVID-19

**DOI:** 10.1590/0034-7167-2024-0105

**Published:** 2025-12-15

**Authors:** Luciana da Rocha Cabral, Maria Sandra Andrade, Juliana da Rocha Cabral, Edilma Gomes Rocha Cavalcante, Emanoela Patrícia Gonçalves Dourado, Regina Célia de Oliveira

**Affiliations:** I Universidade de Pernambuco. Recife, Pernambuco, Brazil; II Universidade Regional do Cariri. Crato, Ceará, Brazil

**Keywords:** Vaccination Hesitancy, COVID-19 Vaccines, Health Belief Model, Validation Study, Nursing., Vacilación a la Vacunación, Vacunas contra la COVID-19, Modelo de Creencias sobre la Salud, Estudio de Validación, Enfermería.

## Abstract

**Objectives:**

to develop and validate the content and construct of an instrument for assessing vaccine hesitancy against COVID-19.

**Methods:**

methodological study, developed in three stages: i) construction of the instrument; ii) content validation by judges; and iii) validation of the construct by the target audience. The Health Beliefs Model was used to develop the instrument.

**Results:**

the instrument was constructed with 45 items. Seven items were excluded because they had a content validity coefficient of less than 0.8. Exploratory and confirmatory factor analyses were carried out to validate the construct. The Kaiser-Meyer-Olkin was 0.801 and Bartlett’s test of sphericity showed p<0.001. Six items did not achieve sufficient factor loading to remain in the instrument. The confirmatory factor analysis revealed a valid and reliable instrument.

**Conclusions:**

the developed instrument shows evidence of validity and is therefore recommended for research aiming to assess the reasons for COVID-19 vaccine hesitancy.

## RESUMEN

**Objetivos:**

construir y validar el contenido y el constructo de un instrumento para evaluar la vacilación a la vacunación del COVID-19.

**Métodos:**

estudio metodológico desarrollado en tres etapas: i) construcción del instrumento; ii) validación del contenido por jueces; e iii) validación del constructo por el público-objeto. El Modelo de Creencias sobre Salud respaldó la elaboración del instrumento.

**Resultados:**

el instrumento se construyó con 45 elementos. Se excluyeron siete por presentar coeficiente de validez de contenido inferior a 0,8. Para la validación del constructo, se realizaron análisis factoriales, exploratorio y confirmatorio. El Kaiser-Meyer-Olkin fue de 0,801 y la prueba de esfericidad de Bartlett, p<0,001. Seis elementos no alcanzaron carga factorial suficiente para permanecer en el instrumento. El análisis factorial confirmatorio mostró un instrumento válido y fidedigno.

**Conclusiones:**

el instrumento construido demuestra evidencia de validez, recomendándose para investigaciones que busquen evaluar los motivos para la vacilación a la vacunación contra la COVID-19.

## INTRODUCTION

Immunization is the most successful public health intervention because it can prevent the development of dozens of infectious diseases, saving millions of lives every year^(1)^. For this reason, the initial fight against the Coronavirus Disease 2019 (COVID-19) pandemic has prompted major efforts by laboratories and research centers to produce immunizers capable of reducing or eliminating the pathogenicity of the new coronavirus^(2)^.

In Brazil, application of the vaccine began in January 2021, through a National Vaccination Operationalization Plan^(3)^. However, despite the positive response to the immunizer evidenced by the health indicators related to COVID-19, until the last week of April 2025, monovalent vaccination coverage (VC) was 86.57% for people with two doses and 19.60% for four doses^(4)^. The high rate of vaccine hesitancy was strengthened by false news spread on digital platforms and anti-vaccination activist campaigns that took place in several countries^(5)^.

Vaccine hesitancy is understood as the delay in accepting or refusing vaccination, despite the availability of immunization services^(6)^. Due to its impact on public health, vaccine hesitancy was listed in 2019 by the World Health Organization as one of the top ten threats to global health^(7)^.

The Health Beliefs Model, which relates aspects of human behavior to individual health choices and underpins the construction of this study’s instrument, can help identify the factors that influence vaccine hesitancy. According to this model, the adoption of preventive behaviors depends on four main perceptions: perceived susceptibility, which refers to the individual’s belief about the likelihood of contracting a disease; perceived severity, which involves the assessment of the severity and possible negative consequences of the disease in one’s life; perceived benefits, which correspond to the conviction that preventive action - in this case, vaccination - will reduce the risks or severity of the disease; and perceived barriers, which are the obstacles, real or perceived, that hinder or discourage the adoption of preventive behavior. Thus, for the decision to be favorable to vaccination, it is necessary for the individual to recognize their vulnerability to illness, understand the Severity of COVID-19, believe in the efficacy of the vaccine and perceive that the advantages of vaccination outweigh the possible barriers^(8)^.

Considering the lack of validated Brazilian instruments for assessing vaccine hesitancy against COVID-19 and the limited number of such instruments available in the international literature^(9,10)^, we proposed the construction of an instrument based on the Health Beliefs Model, capable of correlating sociodemographic data and health conditions with facilitating aspects and barriers to vaccination. The choice of this model is justified by its emphasis on individual risk perception, belief in the severity of the disease, benefits of preventive action and perceived barriers - central factors in the decision to adopt preventive behaviors. In addition, the Health Beliefs Model focuses on individual cognitive perceptions, in line with the profile of the study’s target audience, made up of adults attending primary care services.

In this context, it is necessary to develop new tools that provide a solid conceptual basis for understanding vaccine hesitancy against COVID-19, allowing us to plan strategic interventions that contribute to achieving adequate vaccination coverage.

## OBJECTIVES

To develop and validate the content and construct of an instrument for assessing vaccine hesitancy against COVID-19.

## METHODS

### Ethical aspects

The study was conducted after approval by the Research Ethics Committee of the Hospital Complex of the Oswaldo Cruz University Hospital/University Cardiology Emergency Room of Pernambuco Prof. Luiz Tavares. The Declaration of Free and Informed Consent was signed electronically by the participants in the online survey and in writing for those who responded in person.

### Study design, period and location

This is a methodological study with a quantitative approach, reported in accordance with the recommendations of the EQUATOR network’s Guidelines for Reporting Reliability and Agreement Studies^(11)^.

To carry out the study, psychometric procedures recommended by Pasquali were adopted, through the construction, content validation and construct validation of an instrument to assess vaccine hesitancy against COVID-19, identified by the acronym IHVCOVID-19, carried out from April 2022 to May 2023^(12-14)^.

### Population and sample; inclusion and exclusion criteria

For content validation, the participation of six to 20 judges is recommended^(14)^. The judges were selected using the snowball sampling technique, which allows a sample to be defined by reference^(15)^, based on the following inclusion criteria: a) Graduation from a health course; b) *Stricto Sensu or Lato Sensu* specialization in public health and/or public health and/or infectology and/or family health; c) At least three years’ experience in the field of public health and/or public health and/or vaccination. First, the state coordinator of the immunization program was identified and contacted by telephone. She participated as the first judge and then the other judges were nominated. Judges who met the eligibility criteria and agreed to participate were included in the study.

For construct validation, a minimum of 100 participants is recommended, or five times the number of items analyzed^(16)^. The construct structure was analyzed by the general population using the Google forms platform, with the link disseminated via WhatsApp and email, using the snowball sampling technique^(15)^, in which each participant is encouraged to indicate new respondents. Individuals of any gender, literate and aged ≥ 18 years were included. The initial dissemination of the form took place in the WhatsApp research group of one of the authors of the study.

### Study protocol

The theoretical basis for constructing the tool was based on an integrative review, aimed at defining concepts^(17)^, following the stages of identifying the research question, defining inclusion and exclusion criteria, searching databases, selecting and analyzing studies, interpreting the results and synthesizing the knowledge produced. From this process, the essential elements with the greatest contribution to vaccine hesitancy were extracted: lack of confidence in the COVID-19 vaccine and/or the healthcare team^(18-24)^; belief that COVID-19 is not a dangerous disease^(18,25,26)^; fear of an adverse reaction to the COVID-19 vaccine^(18,21,23,27)^; refusal to receive other vaccines^(18,19,24,28)^; having chronic diseases^(20,28)^; poor access to information about COVID-19 vaccination^(20,23,26)^; sociodemographic conditions^(18-26,28)^; family influence on COVID-19 vaccine hesitancy^(19,20,27)^; and political orientation^(18,21,27)^.

The material was then organized into two sections: the first, called “health conditions related to COVID-19”, included the participants’ sociodemographic data, questions related to the assessment of health conditions, history of coronavirus illness, COVID-19 vaccination schedule and preference for the manufacturer laboratory. The second section, entitled “assessment of vaccine hesitancy against COVID-19”, was composed, a priori, of four domains - susceptibility, Severity, perceived benefits and perceived barriers - with answers distributed on a Likert-type scale: never (1), almost never (2), sometimes (3), almost always (4) and always (5). The first version of the instrument was prepared with a total of 45 items, aligned with the Health Beliefs Model, the theoretical framework adopted.

Fourteen judges were invited. Participation took place remotely, due to incompatible schedules or living outside the state of Pernambuco. All meetings were scheduled in advance. The face-to-face meetings were held at the judges’ place of work, in a private room. The remote meetings took place via the Google Meet platform and the instrument was applied in real time. All meetings were scheduled in advance, held individually and lasted approximately one hour and 30 minutes. During the evaluation of the instrument, the judges were able to read and analyze the material and interact with the researcher whenever necessary.

The items and domains were listed so that the judges could check whether there was agreement on the item within the selected domain. In order to analyze whether the items were understandable to the target population and representative for achieving the objective, each item was evaluated in terms of the criteria of clarity of language, theoretical relevance and practical pertinence using a Likert-type scale, scored from one to five: very little (1), little (2), medium (3), a lot (4), very much (5)^(13)^. A space was left in each item for the judges to make suggestions and/or changes.

After content validation, the instrument was factor analyzed to check its psychometric properties related to construct validity^(16)^.

### Analysis of results and statistics

To validate the content, an analysis of agreement between the judges was carried out and the answers were tabulated in a Microsoft Excel^®^ spreadsheet. The frequency distribution measure was used to characterize the participants. To analyze the data obtained from the IHVCOVID-19, the Content Validity Coefficient (CVC) was used, through which the judges assessed the degree of clarity, pertinence and relevance of each item in the instrument, assigning scores from 1 to 5. A score greater than or equal to 0.80 was adopted as the criterion for satisfactory agreement between the judges, resulting in the decision to keep the item^(29)^.

To validate the construct of the instrument, Exploratory Factor Analysis (EFA) was carried out, using the principal axis factorization model, and Confirmatory Factor Analysis (CFA). Through EFA, it was possible to identify the best underlying relationships between the measured variables, grouping the existing correlations of the variables into factors, which allowed the most representative groupings to be recognized and even the creation of a new set of variables smaller than the original^(16)^. On the other hand, CFA is used to test hypotheses and confirm the extent to which certain variables are representative of a dimension, according to the theoretical basis^(30)^.

To carry out the EFA, sample adequacy was assessed using the Kaiser-Meyer-Olkin (KMO) index, requiring a value equal to or greater than 0.70. To check the adequacy of the sample per item, the Measure of Sampling Adequacy (MSA) was analyzed, with a minimum value of 0.50 required for each item^(16)^.

In order to proceed with the factor analysis, it was necessary to rule out the null hypothesis. To do this, Bartlett’s Test of Sphericity was applied, the aim of which is to analyze the anti-image matrix, refuting the hypothesis that the correlation matrix is an identity matrix. This test is significant when the value is less than 0.05^(16,30)^.

In the EFA, the factor loading of each item was extracted, which represents the correlation between the indicator and the extracted factor. The cut-off point for excluding the item was values below 0.30^(16)^.

With regard to the CFA, the Structural Equation Modeling (SEM) principle was used to confirm the convergent validity and reliability of the instrument, as measured by the composite reliability (CR), which should be above 0.70, and the average variance extracted (AVE), which should be above 0.50. Finally, we calculated the root mean square error of approximation (RMSEA), which must be less than 0.08, and the goodness of fit index (GFI) and adjusted goodness of fit index (AGFI), which must be greater than 0.90^(16)^.

JASP (Jeffrey’s Amazing Statistics Program) and SPSS^®^ (Statistical Package for Social Science) version 26.0 software were used to carry out the statistical analysis of the construct validation.

## RESULTS

The instrument initially constructed resulted in 45 items: ten for health conditions related to COVID-19 and 35 for assessing vaccine hesitancy against COVID-19, distributed into susceptibility (11 items), Severity (seven items), perceived benefits (six items) and perceived barriers (11 items).

Ten judges took part in the content validation. The majority were female (90.0%), with an average age of 44 years (SD=6.84) ranging from 31 to 51 years. Of the judges, seven (70%) had a degree in nursing. In addition, nine (90%) said they had a stricto sensu specialization, four of whom held a master’s degree (40.0%) and five a doctorate (50.0%). The average length of time working in the area of public health and/or immunization was nine years (SD=6.11), with a minimum of four years and a maximum of 20 years. Judges from Pernambuco, Paraíba, Rio Grande do Sul and Rio de Janeiro took part.

The CVC regarding the clarity, pertinence and relevance of the section “health conditions related to COVID-19” is expressed in Table 1.

**Table 1:** Coefficient of Content Validity of the IHVCOVID-19, according to the criteria of clarity of language, practical relevance and theoretical relevance of the section health conditions related to covid -19, Recife, Pernambuco, Brazil, 2023

	Variables	CVCc^†^	CVCp^*^	CVCr^+^	CVCt^§^
Q01	How do you rate your health?	0.88	0.98	0.94	0.93
Q02	Have you ever had COVID-19?	1.00	1.00	0.94	0.98
Q03	Have you had any symptoms compatible with COVID-19?	1.00	0.98	0.90	0.96
Q04	Have you been tested for COVID-19?	0.92	0.86	0.80	0.86
Q05	Have you received any doses of the COVID-19 vaccine?	0.96	0.98	0.96	0.96
Q06	If so, did you have any reaction?	0.98	1.00	0.96	0.98
Q07	Are you familiar with the laboratories that manufacture COVID-19 vaccines?	0.84	0.84	0.78	0.82
Q08	Is there a preference for any manufacturing laboratory?	0.88	0.80	0.78	0.82
Q09	Date of receiving doses	0.74	0.62	0.56	0.64^‡^
Q10	Have you lost anyone in your family to the new coronavirus?	0.98	0.98	0.98	0.98
	**Mean**	0.92	0.90	0.86	0.89

† CVCc- Content Validity Coefficient - Clarity of Language;

* CVCp- Content Validity Coefficient - Practical Relevance; +CVCr- Content Validity Coefficient - Theoretical Relevance; §CVCt- Total Content Validity Coefficient; ‡Items excluded.

The CVC for the clarity, pertinence and relevance of the section “evaluation of vaccine hesitancy against COVID-19” is expressed in Table 2.

**Table 2:** Content Validity Coefficient of IHVCOVID-19, according to the criteria of clarity of language, practical relevance and theoretical relevance of the section evaluation of vaccine hesitancy against COVID-19, Recife, Pernambuco, Brazil, 2023

	Variables	CVCc^†^	CVCp^*^	CVCr^+^	CVCt^§^
	**Susceptibility**				
Q11	Do you believe you are exposed to contracting COVID-19?	0.76	0.94	0.92	0.87
Q12	Are you afraid of contracting COVID-19?	0.90	0.96	0.98	0.94
Q13	Do you believe that your immunity is capable of fighting off the coronavirus infection on its own, should you fall ill?	0.56	0.74	0.74	0.68^‡^
Q14	If you experience respiratory symptoms (such as flu-like symptoms), do you seek medical attention to rule out or confirm COVID-19?	0.84	0.94	0.92	0.90
Q15	Do you wear or have you worn a mask to protect against COVID-19?	1.00	1.00	1.00	1.00
Q16	Do you or have you practiced social distancing to protect against COVID-19?	1.00	1.00	1.00	1.00
Q17	Have you taken or do you take any self-administered medication to prevent becoming ill with COVID-19?	0.72	0.82	0.74	0.76^‡^
Q18	Do you seek or have you sought medical advice to use any medication to prevent becoming ill with COVID-19?	0.88	0.92	0.90	0.90
Q19	Before the COVID-19 pandemic, did you receive the annual influenza vaccine?	0.88	0.92	0.88	0.89
Q20	Do you go to the health service for preventive appointments (without being sick)?	0.92	0.92	0.92	0.92
Q21	Do you go to the health service to check that your vaccination card is up to date?	0.92	0.98	0.98	0.96
	**Severity**				
Q22	Do you believe that COVID-19 is a dangerous disease?	1.0	1.0	1.0	1.0
Q23	Do you believe that a person infected with the coronavirus is at risk of death?	0.86	0.93	0.93	0.91
Q24	Do you believe that a person who has not been vaccinated against COVID-19 has a greater risk of getting the severe form of the disease than a vaccinated person?	0.98	1.0	1.0	0.99
Q25	Do you believe that a person not vaccinated against COVID-19 has a higher risk of dying than a vaccinated person?	0.96	1.0	1.0	0.98
Q26	Do you believe that if you fall ill with COVID-19, it will have negative consequences for your life?	0.98	1.0	0.98	0.98
Q27	Do you believe that if you fall ill with COVID-19, this illness will have negative financial consequences for your life?	0.74	0.78	0.78	0.76^‡^
Q28	Do you believe that if you fall ill with COVID-19, this illness will have negative emotional consequences for your life?	0.68	0.69	0.67	0.68^‡^
	**Perceived Benefits**				
Q29	Do you believe that vaccines protect children from getting sick?	0.92	0.96	0.96	0.94
Q30	Do you believe that vaccines protect adults against diseases such as hepatitis, tetanus, diphtheria, influenza?	0.84	0.80	0.82	0.82
Q31	Do you believe that the coronavirus vaccine protects against getting sick?	0.88	0.91	0.91	0.90
Q32	Do you believe that even without completing the vaccination schedule, the individual is protected against COVID-19?	0.94	0.93	0.93	0.94
Q33	Do you believe that vaccination has contributed to a reduction in deaths from COVID-19?	0.96	1.00	1.00	0.98
Q34	Do you believe that not receiving the vaccine implies more risks to general health than receiving the vaccine?	0.70	0.82	0.76	0.76^‡^
	**Perceived barriers**				
Q35	Do you find it difficult to access public health services?	0.86	0.93	0.93	0.91
Q36	When you need to use health services, are you well received by the UBS team?	0.92	0.87	0.91	0.90
Q37	Do you find it difficult to access vaccines at your PHC?	0.84	0.96	0.96	0.92
Q38	Do you have a Community Health Worker?	0.90	0.64	0.56	0.70^‡^
Q39	Does anyone in your family forbid you from receiving the COVID-19 vaccine?	0.94	0.98	1.0	0.97
Q40	Does your religious belief interfere with your health decisions (such as whether or not to receive the vaccine)?	0.94	1.00	1.0	0.98
Q41	Does the opinion of politicians (president, deputies, mayors) interfere with your health choices?	0.92	0.87	0.89	0.89
Q42	Do you refuse any vaccine because of the manufacturing laboratory?	0.84	0.91	0.89	0.88
Q43	Are you or were you afraid of receiving the vaccine because of any adverse events?	0.76	0.93	0.89	0.86
Q44	Do you trust the efficacy of the COVID-19 vaccine?	0.90	0.98	1.00	0.96
Q45	Do you trust the storage of vaccines?	0.92	0.89	0.84	0.88
	**Mean**	0.87	0.91	0.87	0.89

† CVCc- Coeficiente de Validade de Conteúdo - Clareza de Linguagem;

* CVCp- Coeficiente de Validade de Conteúdo - Pertinência Prática;

+ CVCr- Coeficiente de Validade de Conteúdo - Relevância Teórica; ^**§**^ *CVCt- Coeficiente de Validade de Conteúdo Total; ‡Itens excluídos.*

*Source: JASP 0.16.1.0*

In addition to calculating the CVC, a qualitative analysis was made of the suggestions written by the judges in the “observation” field at the end of the evaluation of each item. After interpreting the findings, there was a slight change in the wording of some items with the removal of the pronoun “you” and the terms ‘believe’ and “think”, in order to make the sentences more objective, while maintaining the purpose of the instrument. It should be noted that not all the items were changed. There were no item changes in relation to the domain.

After content validation, the instrument went on to construct validation with version two of the instrument, containing 38 items, with nine questions in the “Health Conditions Related to COVID-19” section and 29 questions in the “Evaluation of Vaccine Hesitation against COVID-19” section: Susceptibility (nine items), Severity (five items), Perceived Benefits (five items) and Perceived Barriers (ten items). The four domains of the “Evaluation of Vaccine Hesitation against COVID-19” section are anchored in the Health Beliefs Model. As the items in the “Health Conditions Related to COVID-19” section are merely descriptive, they were not subjected to construct validation.

225 people took part in the construct validation, of whom 187 (83.1%) were female, with a predominance of people aged between 30 and 40 (55.5%). Of the participants, 133 (59.1%) said they had higher education and 128 (56.8%) said they earned up to 3 minimum wages.

Corroborating the suitability of the sample for factor analysis, the calculation of the KMO measure obtained a coefficient of 0.801. Bartlett’s test of sphericity reached statistical significance (p < 0.001), refuting the hypothesis of a matrix-identity. All the items in the instrument had an MSA above the recommended level to remain in the study.

**Figure 1:**
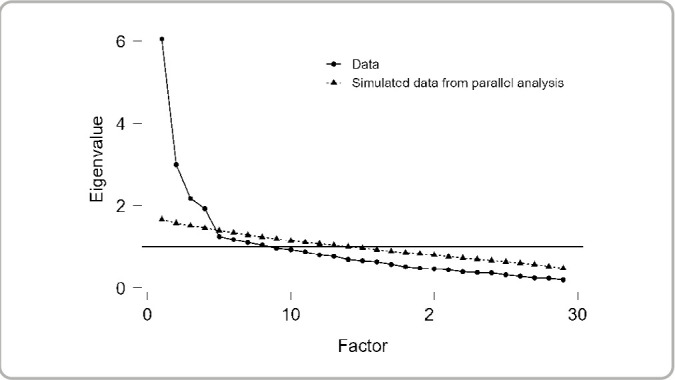
Graphical representation of the scree plot for choosing the number of factors in the instrument for assessing vaccine hesitancy against COVID-19, Recife, Pernambuco, Brazil, 2023

The factor analysis generated the scree plot (Figure 1), which helped decide on the number of factors (domains). This decision was made by comparing the pairwise criteria, based on the number of Eigenvalues equal to or greater than 1.0, which indicates four factors, as well as the Cattell criterion, based on the elbow point of the variance distribution figure.

The EFA indicated four factors (F). However, in order to clarify the understanding of the items that remained in each factor, the factor analysis process contributed to the renaming of the dimensions. Thus, the “Severity” dimension was renamed “Perceived Barriers - Complacency (F4)” and the “Perceived Barriers” dimension became “Perceived Barriers - Beliefs and Attitudes (F3)”. The Perceived Benefits (F1) and Susceptibility (F2) dimensions remained unchanged.

Also in the EFA process, items (Q11, Q14, Q15, Q26, Q32, Q43) were excluded as they had factor loadings < 0.3. It should also be noted that items (Q22: 0.559 and Q23: 0.528) had the highest factor loadings in the Susceptibility factor, while items (Q44: 0.648 and Q45: 0.477) had the highest factor loadings in the “Perceived Benefits” factor, so it was decided to move this item to the factor with the highest factor loading.

We then proceeded with the confirmatory factor analysis, through which it was possible to affirm that the instrument has sufficient indicative CR and AVE values to consider it valid and reliable, as shown in Table 3. Finally, the RMSEA revealed a value of 0.07, the GFI of 0.97 and the AGFI of 0.96, all within the recommended range.

**Table 3:** Confirmatory Factor Analysis of IHVCOVID-19, according to composite reliability criteria and average variance extracted, Recife, Pernambuco, Brazil, 2023

Factor	CR^*^	AVE†
Perceived Benefits (F1)	0.99	0.96
Susceptibility (F2)	0.99	0.92
Perceived Barriers - Beliefs and Attitudes (F3)	0.98	0.94
Perceived Barriers - Complacency (F4)	0.84	0.89

* *CR: Composite Reliability; †AVE: Average Variance Extracted.*

The final instrument included 09 items in the “Health conditions related to COVID-19” section and 23 items in the “Evaluation of Vaccine Hesitation against COVID-19” section, divided into four factors: Perceived Benefits (06 items), Susceptibility (08 items), Perceived Barriers - Beliefs and Attitudes (07 items) and Perceived Barriers - Health Services (02 items). It can be presented in oral or written form and can be filled in by oneself or by the researcher/applicator.

## DISCUSSION

Acceptance of receiving the vaccine against an infectious disease is recognized as the main strategy capable of controlling its spread and ensuring the success of immunization programs^(31)^. In Brazil, the Unified Health System guarantees free access to vaccination against COVID-19, distributed in Primary Care Center.

In the last three years, there has been a significant increase in studies exploring factors associated with vaccine hesitancy against COVID-19 in various population groups and in all countries of the world. However, it has not yet been possible to provide unequivocal evidence on the conditions that interfere with vaccine adherence^(32,33)^.

Thus, following the steps recommended by Pasquali^(12-14)^: the theoretical, the empirical (experimental) and the analytical (statistical) and anchored in a health theory, it was possible to obtain a scientific model capable of contemplating sociodemographic data with the intention of immunization, the perceptions of safety and efficacy of the vaccine, acceptance and trust of the immunizer, anxieties about side effects and selective hesitation due to brand preference. In addition, we sought to identify the barriers that contribute to refusing or delaying vaccination. A validated scientific instrument guarantees scientific backing to generate predictions based on the construction of hypotheses, which allows, through its implementation, to guide actions directed at the population, in order to facilitate communication, intervention and the achievement of the expected results^(34)^.

Content validation resulted in the exclusion of seven questions and revealed agreement between the judges about the appropriate theoretical relevance, practical pertinence and clarity of the language of the instrument. However, in order to favor clarity and objectivity, as well as improving comprehension by all strata of the population who will answer the questionnaire, some items had to be reformulated. All suggestions were accepted at this stage of the research. The process of validation by judges allowed a critical assessment to be carried out by a group of experts with experience in the subject area of the instrument, with the aim of improving the proposed content, making it more precise, clear, reliable and valid for what it is intended to analyze^(13)^.

With regard to construct validation, the removal of items is based on the decision to make the instrument leaner, with greater stability, to facilitate replication in future studies. Furthermore, these removals allow for better refinement in the CFA^(35)^.

The EFA also made it possible to recognize cross-loadings by bifactor structure. This is due to the possibility of assessing the size of the effect of the factors on the items, so it is feasible to allocate the items to other factors because they have higher loadings^(35)^. There was a change in the nomenclature of two dimensions, which may be due to the instrument’s objectivity^(34)^.

The analysis of the main components of this instrument also showed an explained variance per factor of 18.9%, 8.2%, 5.5% and 4.5%, respectively, and a total explained variance of 37.1%.

With regard to the CFA, the instrument showed satisfactory levels of composite reliability and average variance, thus meeting all the criteria and being considered valid. The use of a validated instrument in clinical practice brings clinical benefits and security in relation to the instrument’s reliability for situational diagnostic processes^(36)^.

With regard to vaccination, nursing is the professional category responsible for managing and operating vaccine rooms. For this reason, the use of an unprecedented and valid tool will help to identify the variables of health beliefs that contribute to vaccine hesitancy due to COVID-19. This analysis is crucial for planning strategic actions that favor adherence to this vaccine and, consequently, improve health indicators^(37)^.

There are few instruments aimed at measuring the factors that lead to COVID-19 vaccine hesitancy, despite current epidemiological data showing low CV^(9,10)^. The IHVCOVID-19 aims to fill the gaps in understanding the predictive causes of vaccine hesitancy. For issues of social relevance, such as vaccine hesitancy, the literature points out that it is interesting to have multiple instruments in order to achieve better, clearer answers for planning more targeted actions to solve the problem^(38)^.

### Study limitations

Although the development and validation of this instrument is based on a robust methodology, this research has some limitations. Firstly, all the data used for construct validation was collected cross-sectionally by self-report, which can generate information bias. Secondly, while the snowball sampling technique may introduce biases, attention to geographical diversity and the clear definition of inclusion criteria help to maintain the representativeness of the sample. It is recommended that new studies be carried out with larger samples in order to standardize the scores of the validated instrument.

### Contributions to the field

It should be proposed that this tool be used by nurses in Primary Care Center, with a view to recognizing the population’s health beliefs about immunization against COVID-19. It is worth noting that, in Brazil, nursing is the professional category responsible for the vaccination process at all stages at the local health level. Therefore, this instrument will contribute in an innovative way to the advancement of health and nursing practices in the field of health surveillance.

## CONCLUSIONS

The construction of the instrument followed the steps recommended in the literature for defining the domains and constructing the items. In the content validation stage, substantial changes were made to the items during the evaluation process by the judges. In the end, content validity was achieved to a satisfactory standard, in addition to meeting the parameters recommended in the literature.

As for construct validation, the instrument proved to be valid and accurate for the population studied. Thus, it is possible to attest that the questionnaire developed presents evidence of validity and is therefore recommended for research that seeks to assess the reasons that negatively influence adherence to the COVID-19 vaccine.

It is hoped that this instrument can help to identify the reasons why people miss out on vaccination opportunities. Despite the speed with which lives have been taken by COVID-19, there are still people who have not completed the primary COVID-19 immunization schedule. Identifying the sources of COVID-19 vaccine hesitancy provides a benchmark for interventions aimed at improving vaccination rates.

## Data Availability

The research data are available within the article.
