# Supplementary material for: Development and validation of an instrument to verify vaccination hesitancy against COVID-19
Source: Rev Bras Enferm. 2025 Dec 15;78(5):e20240105. doi: 10.1590/0034-7167-2024-0105 (PMC12707851; doi:10.1590/0034-7167-2024-0105)
Supplement: Translation

# Translation: Construção e validação de um instrumento para verificação da hesitação vacinal contra a COVID-19

**DOI:** 10.1590/0034-7167-2024-0105pt

**Authors:** Luciana da Rocha Cabral, Maria Sandra Andrade, Juliana da Rocha Cabral, Edilma Gomes Rocha Cavalcante, Emanoela Patrícia Gonçalves Dourado, Regina Célia de Oliveira

**Affiliations:** I Universidade de Pernambuco. Recife, Pernambuco, Brasil; II Universidade Regional do Cariri. Crato, Ceará, Brasil

**Keywords:** Hesitação Vacinal, Vacinas contra COVID-19, Modelo de Crenças de Saúde, Estudo de Validação, Enfermagem.

## Abstract

**Objetivos:**

construir e validar o conteúdo e o constructo de um instrumento para avaliação da hesitação vacinal contra a COVID-19.

**Métodos:**

estudo metodológico, desenvolvido em três etapas: i) construção do instrumento; ii) validação de conteúdo por juízes; e iii) validação de constructo pelo público-alvo. O Modelo de Crenças em Saúde embasou a elaboração do instrumento.

**Resultados:**

o instrumento foi construído com 45 itens. Sete itens foram excluídos por apresentarem coeficiente de validade de conteúdo inferior a 0,8. Para a validação de constructo, realizou-se análises fatoriais exploratória e confirmatória. O *Kaiser-Meyer-Olkin* foi de 0,801 e o teste de esfericidade de Bartlett apresentou p<0,001. Seis itens não atingiram carga fatorial suficiente para permanência no instrumento. A análise fatorial confirmatória revelou um instrumento válido e fidedigno.

**Conclusões:**

o instrumento construído apresenta evidência de validade, sendo, portanto, recomendado para pesquisas que busquem avaliar os motivos para hesitação vacinal contra a COVID-19.

## INTRODUÇÃO

A imunização é a intervenção de saúde pública de maior sucesso, por ser capaz de prevenir o desenvolvimento de dezenas de doenças infectocontagiosas, salvando milhões de vidas todos os anos^(1)^. Por isso, a luta inicial contra a pandemia da *Coronavirus Disease 2019* (COVID-19) suscitou grandes esforços dos laboratórios e centros de pesquisas para a produção de imunizantes capazes de reduzir ou eliminar a patogenicidade do novo coronavírus^(2)^.

No Brasil, a aplicação da vacina foi iniciada em janeiro de 2021, por meio de um Plano Nacional de Operacionalização da Vacinação^(3)^. Entretanto, apesar da resposta positiva no imunizante evidenciada pelos indicadores de saúde relacionados à COVID-19, até a última semana de abril de 2025, a cobertura vacinal (CV) monovalente foi de 86,57% para pessoas com duas doses e 19,60% para quatro doses^(4)^. A elevada taxa de hesitação vacinal foi fortalecida por falsas notícias disseminadas nas plataformas digitais e campanhas ativistas antivacinação que ocorreram em vários países^(5)^.

A hesitação vacinal é entendida como o atraso na aceitação ou recusa da vacinação, apesar da disponibilidade nos serviços de imunização^(6)^. Em razão do impacto na saúde pública, a hesitação vacinal foi listada, no ano de 2019, pela Organização Mundial da Saúde como uma das dez principais ameaças à saúde global^(7)^.

O Modelo de Crenças em Saúde, que relaciona aspectos do comportamento humano às escolhas individuais de saúde e fundamenta a construção do instrumento deste estudo, pode contribuir para a identificação dos fatores que influenciam a hesitação vacinal. Segundo esse modelo, a adoção de comportamentos preventivos depende de quatro percepções principais: susceptibilidade percebida, que se refere à crença do indivíduo sobre a probabilidade de contrair uma doença; gravidade percebida, que envolve a avaliação da severidade e das possíveis consequências negativas da doença na sua vida; benefícios percebidos, que correspondem à convicção de que a ação preventiva - neste caso, a vacinação - reduzirá os riscos ou a gravidade da doença; e barreiras percebidas, que são os obstáculos, reais ou percebidos, que dificultam ou desestimulam a adoção do comportamento preventivo. Assim, para que a decisão seja favorável à vacinação, é necessário que o indivíduo reconheça sua vulnerabilidade ao adoecimento, compreenda a gravidade da COVID-19, acredite na eficácia da vacina e perceba que as vantagens da vacinação superam as possíveis barreiras^(8)^.

Considerando a inexistência de instrumentos brasileiros validados para avaliação da hesitação vacinal contra a COVID-19 e o número limitado de desses instrumentos disponíveis na literatura internacional^(9,10)^, foi proposta a construção de um instrumento fundamentado no Modelo de Crenças em Saúde, capaz de correlacionar dados sociodemográficos e condições de saúde aos aspectos facilitadores e às barreiras para a vacinação. A escolha deste modelo justifica-se por sua ênfase na percepção individual de risco, crença na gravidade da doença, benefícios da ação preventiva e barreiras percebidas - fatores centrais na decisão de adoção de comportamentos preventivos. Além disso, o Modelo de Crenças em Saúde foca nas percepções cognitivas individuais, alinhando-se ao perfil do público-alvo do estudo, composto por adultos atendidos nos serviços de atenção primária.

Nesse contexto, torna-se necessária a elaboração de novos instrumentos que forneçam uma base conceitual sólida para o entendimento da hesitação vacinal contra a COVID-19, permitindo o direcionamento do planejamento de intervenções estratégicas que contribuam para o alcance de uma cobertura vacinal adequada.

## OBJETIVOS

Construir e validar o conteúdo e o constructo de um instrumento para avaliação da hesitação vacinal contra a COVID-19.

## MÉTODOS

### Aspectos éticos

O estudo foi conduzido após aprovação pelo Comitê de Ética em Pesquisa do Complexo Hospitalar do Hospital Universitário Oswaldo Cruz/Pronto-Socorro Cardiológico Universitário de Pernambuco Prof.º Luiz Tavares. A Declaração de Consentimento Livre e Esclarecido foi assinada eletronicamente pelos participantes da pesquisa na modalidade on-line e escrito para os que responderam de maneira presencial.

### Desenho, período e local do estudo

Trata-se de um estudo metodológico de abordagem quantitativa reportado conforme as recomendações da ferramenta *Guidelines for Reporting Reliability and Agreement Studies* da rede EQUATOR^(11)^.

Para execução do estudo, adotou-se procedimentos psicométricos preconizados por Pasquali, por meio da construção, validação de conteúdo e validação de constructo de um instrumento para avaliação da hesitação vacinal contra a COVID-19, identificado pela sigla *IHVCOVID-19*, realizado no período de abril de 2022 a maio de 2023^(12-14)^.

### População e amostra; critérios de inclusão e exclusão

Para a validação de conteúdo, recomenda-se a participação de seis a 20 juízes^(14)^. A seleção dos juízes foi conduzida mediante a técnica de amostragem *snowball* ou método bola de neve, que permite a definição de amostra por referência^(15)^, a partir do seguintes critérios de inclusão: a) Possuir graduação em curso da área de saúde; b) Possuir especialização Stricto Sensu ou Lato Sensu em saúde coletiva e/ou saúde pública e/ou infectologia e/ou saúde da família; c) Possuir no mínimo três anos de experiência na área de saúde coletiva e/ou saúde pública e/ou vacinação. Primeiramente, fez-se a identificação e contato telefônico da coordenadora estadual do programa de imunização, que participou como a primeira juíza e seguiu com a indicação dos demais juízes. Foram incluídos na pesquisa os juízes que atendiam aos critérios de elegibilidade e aceitaram participar.

Para a validação de constructo, recomenda-se um mínimo de 100 participantes, ou cinco vezes o número de itens analisados^(16)^. A análise da estrutura do constructo foi realizada pela população geral, por meio da plataforma *Google forms*, com o link divulgado via *WhatsApp* e e-mail, utilizando a técnica de amostragem *snowball* ^(15)^, na qual cada participante é incentivado a indicar novos respondentes. Foram incluídos indivíduos de qualquer gênero, alfabetizado e com idades ≥ 18 anos. A divulgação inicial do formulário ocorreu no grupo de *WhatsApp* de pesquisa de uma das autoras do estudo.

### Protocolo do estudo

A base teórica para a construção do instrumento fundamentou-se em uma revisão integrativa, direcionada para a definição de conceitos^(17)^, seguindo as etapas de identificação da questão de pesquisa, definição dos critérios de inclusão e exclusão, busca nas bases de dados, seleção e análise dos estudos, interpretação dos resultados e síntese do conhecimento produzido. A partir desse processo, foram extraídos os elementos essenciais com maior contribuição para a hesitação vacinal: falta de confiança na vacina contra a COVID-19 e/ou equipe de saúde^(18-24)^; acreditar que a COVID-19 não é uma doença perigosa^(18,25,26)^; medo da reação adversa da vacina contra a COVID-19^(18,21,23,27)^; recusar o recebimento de outras vacinas^(18,19,24,28)^; ser portador de doenças crônicas^(20,28)^; baixo acesso à informação acerca da vacinação contra a COVID-19^(20,23,26)^; condições sociodemográficas^(18-26,28)^; influência familiar para hesitação vacinal contra a COVID-19^(19,20,27)^; e orientação política^(18,21,27)^.

A partir de então, o material construído foi organizado em duas seções: a primeira, denominada “condições de saúde relacionadas à COVID-19”, incluiu dados sociodemográficos dos participantes, questões relacionadas à avaliação da condição de saúde, histórico de adoecimento pelo coronavírus, esquema vacinal contra a COVID-19 e preferência por laboratório fabricante. A segunda seção, intitulada “avaliação da hesitação vacinal contra a COVID-19”, foi composta, a priori, por quatro domínios - susceptibilidade, seriedade, benefícios percebidos e barreiras percebidas - com respostas distribuídas em uma escala do tipo Likert: nunca (1), quase nunca (2), às vezes (3), quase sempre (4) e sempre (5). A primeira versão do instrumento foi elaborada com um total de 45 itens, alinhados ao Modelo de Crenças em Saúde, referencial teórico adotado.

Convidou-se 14 juízes. A participação ocorreu remotamente, por incompatibilidade de agenda ou por residir fora do estado de Pernambuco. Todos os encontros foram agendados previamente. Os encontros presenciais foram realizados no local de trabalho dos juízes, em sala reservada. Enquanto isso, os encontros remotos ocorreram pela plataforma *Google Meet*, com aplicação do instrumento em tempo real. Todos os encontros foram previamente agendados, realizados de forma individual e tiveram duração aproximada de uma hora e 30 minutos. Durante a avaliação do instrumento, os juízes puderam realizar a leitura e análise do material e interagir com o pesquisador sempre que necessário.

Elencou-se os itens e os domínios para que os juízes verificassem se havia concordância do item dentro do domínio selecionado. Para analisar se os itens são compreensíveis à população-alvo e representativos para alcance do objetivo, cada item foi avaliado quanto aos critérios de clareza de linguagem, relevância teórica e pertinência prática por meio uma escala do tipo Likert, pontuado de um a cinco, sendo: pouquíssima (1), pouco (2), média (3), muita (4), muitíssima (5)^(13)^. Em cada item, deixou-se um espaço para inclusão de sugestões e/ou modificação pelos juízes.

Após a validação de conteúdo, foi realizada a análise fatorial do instrumento para verificar suas propriedades psicométricas relacionadas à validade de constructo^(16)^.

### Análise dos resultados e estatística

Para validação de conteúdo, realizou-se a análise de concordância entre os juízes e as respostas foram tabuladas em uma planilha do *Microsoft Excel* ^®^. Utilizou-se a medida de distribuição de frequência para caracterizar os participantes. Para a análise dos dados obtidos no IHVCOVID-19, usou-se o Coeficiente de Validade de Conteúdo (CVC), por meio do qual os juízes avaliaram o grau de clareza, pertinência e relevância de cada item do instrumento, atribuindo pontuações de 1 a 5. O valor maior ou igual a 0,80 foi adotado com critério de concordância satisfatório entre os juízes, resultando na decisão de permanência do item^(29)^.

Para a validação de constructo do instrumento, realizou-se a Análise Fatorial Exploratória (AFE), utilizando-se o modelo de fatoração do eixo principal, e a Análise Fatorial Confirmatória (AFC). Por meio da AFE, foi possível identificar as melhores relações subjacentes entre as variáveis medidas, agrupando as correlações existentes das variáveis em fatores, o que permitiu o reconhecimento dos agrupamentos mais representativos e, inclusive, a criação de um novo conjunto de variáveis menor que o original^(16)^. Por outro lado, a AFC é utilizada para testar hipóteses e confirmar em que medida determinadas variáveis são representativas de uma dimensão, conforme embasamento teórico^(30)^.

Para a realização da AFE, avaliou-se a adequação amostral por meio do índice *Kaiser-Meyer-Olkin* (KMO), sendo requerido um valor igual ou superior a 0,70. Para verificar a adequação da amostra por item, procedeu-se com a análise do *Measure of Sampling Adequacy* (MSA), com valor mínimo exigido de 0,50 para cada item^(16)^.

Para prosseguir com a análise fatorial, foi necessário descartar a hipótese nula. Para isso, aplicou-se o Teste de Esfericidade de Bartlett, cujo objetivo é a análise da matriz anti-imagem, refutando a hipótese de que a matriz de correlações é uma matriz-identidade. Esse teste é significativo quando o valor é menor que 0,05^(16,30)^.

Na AFE, foi extraída a carga fatorial de cada item, que representa a correlação entre o indicador e o fator extraído, o ponto de corte para exclusão do item foi de valores menores que 0,30^(16)^.

Com relação à AFC, utilizou-se o princípio da Modelagem de Equações Estruturais (MEE) - cujo objetivo é confirmar a validade convergente e a fidedignidade do instrumento, aferidas a partir da confiabilidade composta (CC), que deve ter valor superior a 0,70 - e da variância média extraída (VME), que deve ter valor acima de 0,50. Por fim, calculou-se os índices da raiz média quadrática dos erros de aproximação (RMSEA), que devem ser inferiores a 0,08, e os índices de qualidade de ajuste (GFI) e de qualidade de ajuste ajustado (AGFI), os quais devem ser superiores a 0,90^(16)^.

Para realização das análises estatística da validação de constructo, utilizou-se os softwares JASP (*Jeffrey’s Amazing Statistics Program*) e SPSS^®^ (*Statistical Package for Social Science*) versão 26.0.

## RESULTADOS

O instrumento inicialmente construído resultou em 45 itens: 10 para condições de saúde relacionadas à COVID-19, e 35 para avaliação da hesitação vacinal contra a COVID-19. A distribuição se deu da seguinte forma: susceptibilidade (11 itens), seriedade (7 itens), benefícios percebidos (6 itens) e barreiras percebidas (11 itens).

Participaram da validação de conteúdo dez juízes. A maioria do sexo feminino (90,0%), com idade média de 44 anos (DP=6,84) variando entre 31 e 51 anos. Dos juízes, sete (70%) eram formados em enfermagem. Além disso, nove (90%) afirmaram ter especialização stricto sensu, sendo quatro mestres (40,0%) e cinco doutores (50,0%). O tempo de atuação na área de saúde coletiva e/ou saúde pública e/ou imunização foi, em média, de nove anos (DP=6,11), com tempo mínimo de quatro anos e o máximo de 20 anos. Participaram juízes de Pernambuco, Paraíba, Rio Grande do Sul e Rio de Janeiro.

O CVC quanto à clareza, pertinência e relevância da seção “condições de saúde relacionadas à COVID-19” está expresso na Tabela 1.

**Tabela 1:** Coeficiente de Validade de Conteúdo do IHVCOVID-19, segundo os critérios de clareza de linguagem, pertinência prática e relevância teórica da seção condições de saúde relacionadas à COVID-19, Recife, Pernambuco, Brasil, 2023

	Variáveis	CVCc^†^	CVCp^*^	CVCr^+^	CVCt^§^
Q01	Como você avalia sua condição de saúde?	0,88	0,98	0,94	0,93
Q02	Você já teve COVID-19?	1,00	1,00	0,94	0,98
Q03	Apresentou algum sintoma compatível com a COVID-19?	1,00	0,98	0,90	0,96
Q04	Realizou algum teste para diagnóstico da COVID-19	0,92	0,86	0,80	0,86
Q05	Você recebeu alguma dose da vacina contra a COVID-19?	0,96	0,98	0,96	0,96
Q06	Caso tenha recebido, apresentou alguma reação?	0,98	1,00	0,96	0,98
Q07	Você conhece os laboratórios fabricantes das vacinas contra a COVID-19?	0,84	0,84	0,78	0,82
Q08	Existe preferência por algum laboratório fabricante?	0,88	0,80	0,78	0,82
Q09	Data do recebimento das doses	0,74	0,62	0,56	0,64^‡^
Q10	Você perdeu alguém da família para o novo coronavírus?	0,98	0,98	0,98	0,98
	**Média**	0,92	0,90	0,86	0,89

† CVCc- Coeficiente de Validade de Conteúdo - Clareza de Linguagem;

* CVCp- Coeficiente de Validade de Conteúdo - Pertinência Prática;

+ CVCr- Coeficiente de Validade de Conteúdo - Relevância Teórica; ^**§**^ *CVCt- Coeficiente de Validade de Conteúdo Total; ‡Itens excluídos.*

O CVC quanto à clareza, pertinência e relevância da seção “avaliação da hesitação vacinal contra a COVID-19” está expresso na Tabela 2.

**Tabela 2:** Coeficiente de Validade de Conteúdo do IHVCOVID-19, segundo os critérios de clareza de linguagem, pertinência prática e relevância teórica da seção avaliação da hesitação vacinal contra a COVID-19, Recife, Pernambuco, Brasil, 2023

	Variáveis	CVCc^†^	CVCp^*^	CVCr^+^	CVCt^§^
	**Susceptibilidade**				
Q11	Você acredita que está exposto a contrair a COVID-19?	0,76	0,94	0,92	0,87
Q12	Você tem medo de contrair a COVID-19?	0,90	0,96	0,98	0,94
Q13	Você acredita que sua imunidade é capaz de combater sozinha a infecção do coronavírus, em caso de adoecimento?	0,56	0,74	0,74	0,68^‡^
Q14	Ao apresentar sintomas respiratórios (como um quadro gripal), você procura atendimento médico para descartar ou confirmar COVID-19?	0,84	0,94	0,92	0,90
Q15	Você utiliza ou utilizou máscara para proteção contra a COVID-19?	1,00	1,00	1,00	1,00
Q16	Você pratica ou praticou o distanciamento social para proteção contra a COVID-19?	1,00	1,00	1,00	1,00
Q17	Você fez ou faz uso de alguma medicação autoadministrada para prevenir o adoecimento pela COVID-19?	0,72	0,82	0,74	0,76^‡^
Q18	Você procura ou procurou orientação médica para fazer uso de alguma medicação com o intuito de prevenir o adoecimento pela COVID-19?	0,88	0,92	0,90	0,90
Q19	Antes da pandemia da COVID-19, você recebeu anualmente a vacina contra a influenza (vacina da gripe)?	0,88	0,92	0,88	0,89
Q20	Você procura o serviço de saúde para realizar consultas preventivas (sem estar doente)?	0,92	0,92	0,92	0,92
Q21	Você consulta o serviço de saúde para verificar se o seu cartão de vacina está atualizado?	0,92	0,98	0,98	0,96
	**Seriedade**				
Q22	Você acredita que a COVID-19 seja uma doença perigosa?	1,0	1,0	1,0	1,0
Q23	Você acredita que uma pessoa infectada pelo coronavírus apresenta risco de morte?	0,86	0,93	0,93	0,91
Q24	Você acredita que uma pessoa não vacinada contra a COVID-19 tem mais risco de ter a forma grave da doença do que uma pessoa vacinada?	0,98	1,0	1,0	0,99
Q25	Você acredita que uma pessoa não vacinada contra a COVID-19 tem mais risco de morrer do que uma pessoa vacinada?	0,96	1,0	1,0	0,98
Q26	Você acredita que caso adoeça pela COVID-19, esse adoecimento trará consequências negativas para a sua vida?	0,98	1,0	0,98	0,98
Q27	Você acredita que caso adoeça pela COVID-19, esse adoecimento trará consequências financeiras negativas para a sua vida?	0,74	0,78	0,78	0,76^‡^
Q28	Você acredita que, caso adoeça pela COVID-19, esse adoecimento trará consequências emocionais negativas para a sua vida?	0,68	0,69	0,67	0,68^‡^
	**Benefícios Percebidos**				
Q29	Você acredita que as vacinas protejam as crianças contra o adoecimento?	0,92	0,96	0,96	0,94
Q30	Você acredita que as vacinas protegem os adultos contra as doenças, tais como hepatite, tétano, difteria, influenza?	0,84	0,80	0,82	0,82
Q31	Você acredita que a vacina do coronavírus protege contra o adoecimento?	0,88	0,91	0,91	0,90
Q32	Você acredita que mesmo sem completar o esquema vacinal, o indivíduo fica protegido contra a COVID-19?	0,94	0,93	0,93	0,94
Q33	Você acredita que a vacinação contribuiu para a redução das mortes pela COVID-19?	0,96	1,00	1,00	0,98
Q34	Você acredita que o não recebimento da vacina implica em mais riscos para a saúde geral do que o recebimento da vacina?	0,70	0,82	0,76	0,76^‡^
	**Barreiras Percebidas**				
Q35	Você encontra dificuldade de acesso aos serviços públicos de saúde?	0,86	0,93	0,93	0,91
Q36	Quando você necessita utilizar os serviços de saúde, é bem recebido pela equipe da UBS?	0,92	0,87	0,91	0,90
Q37	Você encontra dificuldade de acesso às vacinas na sua UBS?	0,84	0,96	0,96	0,92
Q38	Você tem Agente de Saúde?	0,90	0,64	0,56	0,70^‡^
Q39	Você tem alguém da família que o proíbe de receber a vacina contra a COVID-19?	0,94	0,98	1,0	0,97
Q40	A sua crença religiosa interfere nas suas decisões de saúde (como, por exemplo, em receber ou não a vacina)?	0,94	1,00	1,0	0,98
Q41	A opinião de políticos (presidente, deputados, prefeitos) interfere nas suas escolhas de saúde?	0,92	0,87	0,89	0,89
Q42	Você recusa alguma vacina em razão do laboratório fabricante?	0,84	0,91	0,89	0,88
Q43	Você tem ou teve receio de receber a vacina em razão de algum evento adverso?	0,76	0,93	0,89	0,86
Q44	Você confia na eficácia da vacina contra a COVID-19?	0,90	0,98	1,00	0,96
Q45	Você confia no armazenamento das vacinas?	0,92	0,89	0,84	0,88
	**Média**	0,87	0,91	0,87	0,89

† CVCc- Coeficiente de Validade de Conteúdo - Clareza de Linguagem;

* CVCp- Coeficiente de Validade de Conteúdo - Pertinência Prática;

+ CVCr- Coeficiente de Validade de Conteúdo - Relevância Teórica; ^**§**^ *CVCt- Coeficiente de Validade de Conteúdo Total; ‡Itens excluídos.*

*Fonte: JASP 0.16.1.0*

Além do cálculo do CVC, fez-se uma análise qualitativa das sugestões escritas pelos juízes no campo “observação”, ao final da avaliação de cada item. Após a interpretação dos achados, houve pequena mudança na escrita de alguns itens com a retirada do pronome de tratamento “você” e dos termos “acredita” e “acha”, com o intuito de deixar as frases mais objetivas, mantendo a proposta do instrumento. Ressalta-se que não foram todos os itens que sofreram alterações. Não houve mudança de item em relação ao domínio.

Após validação de conteúdo, o instrumento seguiu para validação de constructo com a versão dois do instrumento, contendo 38 itens, sendo 9 questões da seção “Condições de Saúde Relacionadas à COVID-19”, e 29 questões da seção “Avaliação da Hesitação Vacinal contra a COVID-19”: Susceptibilidade (nove itens), Seriedade (cinco itens), Benefícios Percebidos (cinco itens) e Barreiras Percebidas (dez itens). Os quatro domínios da seção “Avaliação da Hesitação Vacinal contra a COVID-19” estão ancorados no Modelo de Crenças em Saúde. Por trazer questões meramente descritivas, os itens da seção “Condições de Saúde Relacionadas à COVID-19” não foram submetidos à validação de constructo.

Participaram da validação de constructo 225 pessoas, das quais 187 (83,1%) eram do sexo feminino, houve predomínio da faixa etária dos 30 a 40 anos (55,5%). Dos participantes, 133 (59,1%) declararam ter ensino superior e 128 (56,8%) afirmaram receber até 3 salários-mínimos.

Corroborando para a adequabilidade da amostra para análise fatorial, o cálculo da medida do KMO obteve coeficiente de 0,801. O teste de esfericidade de Bartlett atingiu significância estatística (p < 0,001), ao refutar a hipótese de uma matriz-identidade. Todos os itens do instrumento apresentaram MSA acima do recomendado para permanência no estudo.

Na realização da Análise Fatorial, gerou-se o *scree plot* (Figura 1), que contribuiu para decisão sobre o número de fatores (domínios). A decisão é tomada a partir da comparação entre os critérios de par, baseado no número de *Eigenvalues* (Valores próprios) iguais ou superiores a 1,0, que indica quatro fatores, assim como o critério de Cattell, baseado no ponto do cotovelo do gráfico de distribuição de variâncias.

**Figura 1:** Representação gráfica do *scree plot* para escolha do número de fatores do instrumento de avaliação da hesitação vacinal contra a COVID-19, Recife, Pernambuco, Brasil, 2023

A AFE indicou quatro fatores (F). No entanto, com a finalidade de clarificar o entendimento sobre os itens que permaneceram em cada fator, o processo de análise fatorial contribuiu para a renomeação das dimensões. Sendo assim, a dimensão “Seriedade” passou a ser intitulada “Barreiras Percebidas - Complacência (F4)” e a dimensão “Barreiras Percebidas” tornou-se “Barreiras Percebidas - Crenças e Atitudes (F3)”. As dimensões Benefícios Percebidos (F1) e Susceptibilidade (F2) permaneceram com suas nomenclaturas inalteradas.

Ainda, no processo da AFE, excluiu-se os itens (Q11, Q14, Q15, Q26, Q32, Q43), pois apresentaram cargas fatoriais < 0,3. Destaca-se que os itens (Q22: 0,559 e Q23: 0,528) apresentaram maior carga fatorial no fator Susceptibilidade, enquanto os itens (Q44: 0,648 e Q45: 0,477) apresentaram maior carga fatorial no fator “Benefícios Percebidos”, por isso, decidiu-se pela mudança desse item para o fator com maior carga fatorial.

Prosseguiu-se, então, com a análise fatorial confirmatória, por meio dela, foi possível afirmar que o instrumento possui valores indicativos da CC e da VME suficientes para considerar este instrumento válido e fidedigno, expresso na Tabela 3. Por fim, o RMSEA revelou valor de 0,07, o GFI de 0,97 e o AGFI de 0,96, todos dentro do preconizado.

**Tabela 3:** Análise Fatorial Confirmatória do IHVCOVID-19, segundo critérios de confiabilidade composta e variância média extraída, Recife, Pernambuco, Brasil, 2023

Fator	CC^*^	VME†
Benefícios Percebidos (F1)	0,99	0,96
Susceptibilidade (F2)	0,99	0,92
Barreiras Percebidas - Crenças e Atitudes (F3)	0,98	0,94
Barreiras Percebidas - Complacência (F4)	0,84	0,89

* *CC: Confiabilidade Composta;* **†** *VME: Variância Média Extraída.*

O instrumento final foi contemplado com 09 itens na seção “Condições de saúde relacionadas à COVID-19” e 23 itens na seção “Avaliação da Hesitação Vacinal contra a COVID-19”, dividido em quatro fatores: Benefícios Percebidos (06 itens), Susceptibilidade (08 itens), Barreiras Percebidas - Crenças e Atitudes (07 itens) e Barreiras Percebidas - Serviços de Saúde (02 itens). O mesmo pode ser apresentado na forma oral ou escrita, podendo ser autopreenchido ou preenchido pelo pesquisador/aplicador.

## DISCUSSÃO

A aceitação em receber a vacina contra uma doença infecciosa é reconhecida como a principal estratégia capaz de controlar a sua propagação e garantir o sucesso dos programas de imunização^(31)^. Na realidade brasileira, por meio do Sistema Único de Saúde, é garantido o acesso gratuito à vacinação contra a COVID-19, distribuída nas Unidades Básicas de Saúde.

Nos últimos três anos, houve aumento significativo de estudos explorando fatores associados à hesitação da vacina contra a COVID-19 em diversos grupos populacionais e em todos os países do mundo. No entanto, ainda não foi possível fornecer evidências unívocas sobre as condições que interferem na adesão vacinal^(32,33)^.

Dessa forma, seguindo as etapas preconizadas por Pasquali^(12-14)^: o teórico, o empírico (experimental), o analítico (estatístico) e ancorado em uma teoria de saúde, foi possível a obter um modelo científico capaz de contemplar dados sociodemográficos com a intenção de imunização, as percepções de segurança e eficácia da vacina, aceitação e confiança do imunizante, anseios de efeitos colaterais e hesitação seletiva por preferência de marca. Ademais, buscou-se identificar as barreiras que contribuem para a recusa ou atraso da vacinação. Um instrumento científico validado garante respaldo científico para gerar previsões com base na construção de hipóteses, o que permite, pela sua implementação, nortear as ações direcionadas à população, de modo a facilitar a comunicação, a intervenção e o alcance dos resultados esperados^(34)^.

A validação de conteúdo resultou na exclusão de sete questões e revelou concordância entre os juízes acerca da adequada relevância teórica, pertinência prática e clareza na linguagem do instrumento construído. No entanto, a fim de favorecer a clareza e objetividade, além de melhorar a compreensão por todos os estratos da população que responderá ao questionário, foi requerida a reformulação de alguns itens. Dessa forma, todas as sugestões foram acatadas nesta etapa da pesquisa. O processo de validação por juízes permitiu uma avaliação crítica realizada por um grupo de peritos com experiência na área temática do instrumento, com a finalidade de melhorar o conteúdo proposto, tornando-o mais preciso, claro, confiável e válido naquilo que se propõe a analisar^(13)^.

No que tange à validação de constructo, a retirada dos itens está ancorada na decisão de deixar o instrumento mais enxuto, com maior estabilidade, para facilitar a replicação em estudos futuros. Ademais, as remoções possibilitam um melhor refinamento na AFC^(35)^.

A AFE permitiu, ainda, o reconhecimento de cargas cruzadas por estrutura *bifactor.* Isso acontece em razão da possibilidade de avaliar o tamanho do efeito dos fatores sobre os itens, sendo, portanto, factível alocar os itens para outros fatores, por apresentaram cargas superiores^(35)^. Houve mudança na nomenclatura de duas dimensões, o que pode ocorrer para favorecer a objetividade do instrumento^(34)^.

Na análise dos componentes principais deste instrumento, também observou-se uma variância explicada por fator com valores de 18,9%, 8,2%, 5,5% e 4,5%, respectivamente, e uma variância explicada total de 37,1%.

A respeito da AFC, verifica-se que o instrumento apresentou níveis de confiabilidade composta e variância média satisfatórias, enquadrando-se, assim, em todos os critérios, sendo considerado válido. A utilização na prática clínica de um instrumento validado confere benefícios clínicos e traz segurança em relação à confiabilidade do instrumento para os processos de diagnósticos situacionais^(36)^.

No tocante à vacinação, a enfermagem é a categoria profissional responsável pelo gerenciamento e funcionamento das salas de vacinas. Por isso, a utilização de uma ferramenta inédita e válida irá auxiliar na identificação das variáveis das crenças em saúde que contribuem para a hesitação vacinal da COVID-19. Esta análise é de importância crucial para o planejamento de ações estratégicas que favoreçam a adesão da referida vacina, e, consequentemente, a melhoria dos indicadores de saúde^(37)^.

Existem poucos instrumentos com objetivo de mensurar os fatores que levam à hesitação vacinal da COVID-19, apesar de os dados epidemiológicos atuais revelarem baixa CV^(9,10)^. O IHVCOVID-19 visa preencher as lacunas sobre o entendimento das causas preditoras da hesitação vacinal. Para temas com relevância social, como a hesitação vacinal, a literatura aponta ser interessante a existência de múltiplos instrumentos a fim de alcançar melhores respostas mais claras para o planejamento de ações mais direcionadas à resolução do problema^(38)^.

### Limitações do estudo

Embora o desenvolvimento e a validação deste instrumento se baseiem em uma metodologia robusta, esta pesquisa tem algumas limitações. Primeiro, todos os dados utilizados para a validação de constructo foram coletados de forma transversal por autorrelato, o que pode gerar viés de informação. Em segundo lugar, a técnica de amostragem *snowball* possa introduzir vieses, a atenção à diversidade geográfica e a definição clara de critérios de inclusão contribuem para manter a representatividade da amostra. Recomenda-se a condução de novos estudos com amostras maiores, com objetivo de realizar a normatização dos escores do instrumento validado.

### Contribuições para a área

Propõe-se a utilização deste instrumento por enfermeiros das Unidades Básicas de Saúde, com vistas a reconhecer as crenças de saúde da população acerca da imunização contra a COVID-19. Vale destacar que, no Brasil, a enfermagem é a categoria profissional responsável pelo processo de vacinação em todas as etapas no nível local de saúde. Portanto, este instrumento contribuirá de maneira inovadora para o avanço das práticas em saúde e enfermagem no campo da vigilância em saúde.

## CONCLUSÕES

A construção do instrumento seguiu as etapas preconizadas na literatura para definição dos domínios e construção dos itens. Na etapa de validação de conteúdo, realizou-se mudanças substanciais dos itens no decorrer do processo de avaliação pelos juízes. Ao final, alcançou-se a validade de conteúdo com padrão satisfatório, além de atingir os parâmetros preconizados pela literatura.

Quanto à validação de constructo, demonstrou-se que o instrumento é válido e preciso para esta população estudada. Assim, é possível atestar que o questionário construído apresenta evidência de validade, sendo, portanto, recomendado para pesquisas que busquem avaliar os motivos que influenciam negativamente na adesão à vacina contra a COVID-19.

Espera-se que o instrumento possa contribuir para a identificação das razões que levam as pessoas à perda de oportunidade vacinal. Apesar da velocidade com que vidas foram ceifadas pela COVID-19, ainda existem pessoas que não completaram o esquema primário da imunização contra a doença. Identificar as fontes de hesitação da vacina COVID-19 fornece uma referência para intervenções que objetivam melhorar as taxas de vacinação.

## Data Availability

Os dados da pesquisa estão disponíveis no corpo do artigo.
